# Parent Activation Measure for Developmental Disabilities (PAM-DD) in Caregivers of Individuals With ASD

**DOI:** 10.1007/s10803-021-05263-7

**Published:** 2022-01-20

**Authors:** Yue Yu, Lisa Ruble, John McGrew, Donna Murray

**Affiliations:** 1 Present Address: MIND Institute, University of California, 2825 50th Street, Davis, Sacramento, CA 95817 USA; 2 Present Address: Teachers College, Ball State University, Muncie, IN USA; 3 Department of Psychology, Indiana University Purdue University Indianapolis, Indianapolis, IN USA; 4 Autism Speaks, New York, NY USA; 5 Division of Developmental and Behavioral Pediatrics, Cincinnati Children’s Hospital Medical Center, Columbus, OH USA; 6 Department of Pediatrics, College of Medicine, University of Cincinnati, Columbus, OH USA

**Keywords:** ASD, Parent activation, Caregiver, PAM-DD

## Abstract

Activation refers to patients’ belief, knowledge, ability, and persistence to manage care. The concept is adapted to parent activation in developmental disorders. This study examined the psychometrics of the Parent Activation Measure for Developmental Disabilities (PAM-DD) and factors related to parent activation in ASD. Data from 658 caregivers of children with ASD in the Autism Treatment Network Registry Call Back Assessment study were analyzed. The actual ordering of the scale items was inconsistent with the assumptions of a Guttman scaling. Factor analysis revealed two PAM-DD factors. Lower child symptom severity was related to higher Factor 1 and lower Factor 2 activation. Future studies should use caution when treating PAM-DD as a Guttman and unidimensional scale.

Activation is of interest to researchers and clinicians because of its importance to medical and behavioral health outcomes and its natural fit with consumer driven health services (Hibbard & Greene, 2013; Hibbard et al., 2004). Hibbard et al. (2004) first described activation with respect to patients’ belief, knowledge, ability, and persistence to manage one’s care in the health outcome research. Adult consumers with higher activation engage more frequently and intentionally in evidence-based health-related and preventative behavior practices (Hibbard & Greene, 2013). When engaged with proactive service providers, highly activated individuals with a chronic condition also demonstrate better functional and clinical outcomes (Glasgow et al., 2005; Parchman et al., 2010).

Although most activation research has focused on patient’s activation regarding their own physical health care, recently, there has been interest in applying activation to parents or caregivers of children with chronic physical (Decamp et al., 2016, 2019; Liberman & Pham, 2018) and mental health conditions (Green et al., 2019; Thomas et al., 2017) including autism spectrum disorder (ASD; Ruble et al., 2018, 2019). Renamed Parent Activation, parental activation is particularly relevant for children with chronic, “lifelong” developmental disorders (DD) such as ASD because caregivers must navigate a complex and often challenging service system that begins even before the diagnosis is made and continues throughout the child’s life (Smith et al., 2020).

Parents of children with ASD commonly report access to timely and quality care is uneven, disjointed, and difficult to obtain (Smith et al., 2020). Examples include studies on access to care, that is family-centered, comprehensive, and coordinated, also referred to as a medical home (Brachlow et al., 2007). Data from the National Survey for Children’s Health of 495 children with autism and 18,119 children with other conditions reported that parents of children with autism were 55% less likely to endorse care consistent with a medical home compared to those with other conditions, a finding confirmed by other studies (Carbone et al., 2010). Further, despite the promulgation of best practice guidelines (Zwaigenbaum et al., 2015), pediatricians report a lack of knowledge and training in providing a medical home for ASD (Golnik et al., 2009). These negative experiences have consequences as parents of children with ASD report more dissatisfaction with services, stress, and caregiver strain compared to parents of children with other DD (Hayes & Watson, 2013; Liptak et al., 2006).

To date, few studies have examined activation in parents of children with ASD. Because autism affects as many as 1 out of 54 children (Baio et al., 2018) with an estimated lifetime cost of $3.6 million (Cakir et al., 2020) and annual US costs exceeding $268 billion (Leigh & Du, 2015), the paucity of research on parent activation is lamentable. We could only identify three studies. Two examined associations between parent activation and parent outcomes and the third examined youth outcomes. Highly activated parents reported lower stress, greater satisfaction with interventions, and greater ability to manage child-related issues (Ruble et al., 2018). In a second larger study of 260 parents in the Autism Treatment Network (ATN), Crossman et al. (2020) found that family navigation services were associated with increased parent activation and decreased caregiver strain. Finally, for youth outcomes, in a small randomized control trial with high school age ASD youth, Ruble et al. (2019) found that parent activation was associated with increased youth employment. Although limited, these studies suggest important associations between activation and both caregiver and youth outcomes, and justify further investigation of caregiver activation in ASD.

Currently, studies of parent activation have been based on measures adapted from the original Patient Activation Measure (PAM). As with any instrument, however, investigation of the psychometric properties is essential. A few studies of the parent PAM (P-PAM), originally adapted from the PAM, are available. DeCamp et al., (2016, 2019) analyzed surveys of Spanish and English speaking caregivers using the P-PAM. The P-PAM had good internal consistency and acceptable test–retest reliability. However, factor structure differed from the original PAM. Authors cautioned that associations between health care outcomes and activation found using the original PAM may differ when using the P-PAM. In another study of the P-PAM, using emergency department data, Liberman and Pham (2018) found that the P-PAM failed to adequately fit the data and recommended that before the P-PAM is adopted for use, more research is needed to understand parent activation, its associations with outcomes, and its psychometric properties. A study of another version of the P-PAM adapted for Mental Health (P-PAM-MH), found initial support for reliability and validity of the measure (Green et al., 2019). However, they did not examine the factor structure. Finally, the PAM-DD mentioned above, also adapted from the P-PAM to assess parent activation of children with DD, has not been studied for its psychometric properties (Brannan et al., 1997).

A unique feature of the PAM, the P-PAM, and measures derived from them, is the psychometric theoretical framework. The PAM is based on Guttman scaling or cumulative scaling, and is intended for unidimensional measurement. Items are ordered hierarchically. Statements are arranged so that a rater who agrees with any particular item should also agree with every lower-order item that precedes it. Statement order reflects increasing intensity of attitude along a stepped continuum. The point at which the respondent disagrees reflects the respondent’s scale position with the assumption that all items that follow are also rejected (more difficult or extreme).

Consistent with Guttman scaling, the original PAM has four scoring levels (Hibbard et al., 2004). The lowest level indicates relatively weak levels of activation (a) disengaged and overwhelmed. The second level represents (b) becoming aware but still struggling. The third level represents (c) taking action, and the highest and strongest level of activation denotes (d) maintaining action. The PAM, and scales adapted from it, e.g. the PAM-DD, then, should include items rank ordered in a manner consistent with the original Guttman scaling described above.

Given the potential significance of activation in studies on delivery and outcomes of medical and behavioral health services of parents and children with ASD, more research on the measurement of activation and child, parent, and treatment factors associated with activation are warranted. Moreover, given the prominence of the PAM and activation scales adapted from it, more research on its psychometric properties, factor structure, and presumed Guttman scaling is necessary. Thus, the purpose of this study was to examine (a) the psychometrics of the PAM-DD when used in its original Guttman form and, for comparison, when used as a raw scale, specifically with respect to unidimensionality, factor structure, and internal consistency; and (b) factors related to activation, including demographic, child, parent, and treatment variables cross sectionally.

## Methods

### Study Design

The Autism Speaks Autism Treatment Network (ATN)/Autism Intervention Research Network on Physical Health (AIR-P) is a network consisting of 12 academic medical centers across North America providing clinical services to over 35,000 children with ASD annually and has historical strengths in research, building consensus guidelines, family engagement, and creating toolkits for patients, families, and clinicians. Over 7000 children and adolescents with ASD have been enrolled. For additional information and greater details about the design of the ATN and ATN registry, please refer to prior publications (Murray et al., 2016).

This project is a secondary data analysis of the Registry Call Back Assessment (RCBA) study, which is a substudy of the ATN registry. The goal was to enroll 50 patients from a randomly-generated list of 65 eligible participants at each ATN site who met the inclusion criteria: previous ATN assessment between the years 2011 through 2016 or current enrollment into the ATN Registry; non-missing domain scores for the Vineland Scale (Communication, Socialization, Daily Living Skills); meets DSM-IV criteria for any pervasive developmental disorder, or DSM-5 criteria for ASD; informed consent of parent/guardian; and assent/consent of child (minor) subjects, as required by the governing IRB/REB. Study staff tracked individuals who refused consent or did not respond to multiple contact attempts and attempted to capture reason-for-refusal. Non-identifiable data from the ATN registry study was used to describe the non-participating study population and identify sampling biases (Murray et al., 2016).

The RCBA study assessments were completed either in person, over the phone, or online. To reduce participant burden, standardized assessments (e.g. CBCL, ABC, etc.) collected by other agencies within 6 months of an RCBA study visit were accepted and used for study purposes.

### Measures

*Abberant Behavior Checklist (ABC; *Aman et al., 1985***)*** is a parent-reported questionnaire that assesses challenging behavior among individuals with DD. The ABC-Community form consists of 58 questions and five subscales: (1) Irritability, (2) Social Withdrawal/Lethargy, (3) Stereotypic Behavior, (4) Hyperactivity/Noncompliance, and (5) Inappropriate Speech. Higher scores indicate increased challenging behaviors. The ABC subscales have high internal consistency, good test–retest reliability, and established validity. Only the subscale data were available from the RCBA study.

*Autism Impact Measure (AIM; *Kanne et al., 2014***)*** is a 41-item a parent-reported measure assessing the impact of treatment on a child with ASD. The AIM uses a 2-week recall period with items rated on two 5-point scales for frequency (1 “Never” to 5 “Always”) and impact (1 “Not at all” to 5 “Severely”). Psychometric properties were examined using a subsample (n = 440) of children with ASD enrolled in the ATN. Test–retest reliability, cross-informant reliability, and convergent validity were strong. The total score was used. Higher scores indicate greater treatment impact. The internal consistency reliability in the current study was strong (α = 0.95).

*Caregiver Strain Questionnaire (CGSQ; *Brannan et al., 1997***)*** is a 21-item parent-reported measure of emotional, physical, and financial strain. Items are rated on a 5-point Likert scale, 1 “not at all” to 5 “very much.” Higher scores indicate greater caregiver strain. The mean score was used. The internal consistency reliability in the current study was strong (α = 0.92).

*Child Behavior Checklist (CBCL; *Achenbach, 1987***)*** assesses a wide range of challenging behaviors in children with DSM-5 diagnoses. We also included raw scores from two DSM-IV-oriented scales that changed their scoring algorithm in the update to DSM-5. The various scales are separated into two age defined subgroups, Preschool-age (1½–5) and School-age (6–18). Both versions have multiple subscales and composite scores available. The CBCL and its respective subscales have been shown to have good internal consistency and adequate validity (Pandolfi et al., 2012). The Internalizing Problems and Externalizing Problems scales were used in the current study. Higher scores indicate higher levels of internalizing and externalizing problems. Only the subscale data were available from the RCBA study.

*Children’s Sleep Habits Questionnaire (CSHQ; *Owens et al., 2000***)*** screens for common sleep problems, ages 4–12, based on parent report of occurrence, frequency, and impact of a child’s sleep habits and sleep problems. With the exception of the sleepiness items 32 and 33 (0 = Not sleepy, 1 = Very sleepy, 2 = Falls asleep), questions are rated on a 3-point Likert scale (0 = Rarely, 1 = Sometimes, 2 = Usually). The CSHQ demonstrated fair reliability and validity. The total score was used. Higher scores indicate more sleep problems. The internal consistency reliability in the current study was adequate (α = 0.62).

*Parent Activation Measure for Developmental Disabilities (PAM-DD; *Ruble et al., 2019***)*** was adapted from the *Patient Activation Measure* developed by Hibbard et al. (2004). The 13-item PAM-DD assesses parental motivation and involvement in caring for a child with DD. Items measure caregivers' knowledge and self-efficacy in accessing care and treatment, actions towards care and treatment, and stress and strain. Items are scored using a 4-point Likert scale (1 = disagree strongly; 4 = agree strongly). Raw scores are summed and scaled from 0 to 100 using weights based on Guttman scaling. Because our primary research question concerns the psychometric adequacy of the PAM-DD, we conducted an exploratory factor analysis (EFA) of the item raw scores, which revealed two usable independent PAM-DD factors (see results). Thus, four different parent activation scores were used and compared in the analyses: a total and two factor scores based on raw item scoring and the Guttmann total score. Higher scores correspond to higher activation. Factor subscale scores were calculated by summing item scores assigned to the factor with the highest item-factor weights. The internal consistency for the Guttmann scale was adequate (α = 0.83; Ruble et al., 2019). The internal consistency reliability for the total raw score PAM-DD in the current study was strong (α = 0.89). The internal consistency reliability for factor 1 (α = 0.88) also was strong and was acceptable for factor 2 (α = 0.66).

*Pediatric Quality of Life Inventory (PedsQL; *Varni et al., 1999***)*** is a 23-item parent-reported measure that assesses a child’s health-related quality of life across three areas: social, physical, and mental well-being. Four age-based modules were used: Ages 2–4, 5–7, 8–12, and 13–18. The PedsQL comprises four multidimensional Generic Core Scales: Physical Functioning, Emotional Functioning, Social Functioning, and School Functioning. The total score was used. Higher scores indicate greater quality of life. Only the scales data were available from the RCBA study; therefore, the interrater reliability was not available in this study. The scale has good internal consistency, construct and clinical validity (Varni et al., 1999).

*Vineland Adaptive Behavior Scale II Edition (Vineland-II; *Sparrow et al., 2005***)*** is a semi-structured interview administered to a caregiver to assess a child's adaptive functioning. The Vineland-II consists of five adaptive domains and an Adaptive Behavior Composite score. Items are rated 0, 1, or 2. Lower scores indicate greater impairment. Overall Adaptive Behavior Composite was used in this study. Only the domains data were available from the RCBA study; therefore, the interrater reliability was not available in this study. The Vineland-II has been shown to have good test–retest reliability and acceptable levels of internal consistency (Sparrow et al., 2005).

*Parent Visit Assessment* was developed for the ATN Registry study to collect demographic information (e.g. caregiver gender, parental education) and three child related variable subscales (i.e. parent concerns, number of therapies received, comorbidities). The 15-item parental concerns are rated as yes or no (0 = no, 1 = yes; e.g. anxiety, GI problems). The total number of parental concerns was summed to create a subscale. Internal consistency reliability was adequate in the current study (α = 0.72). High scores indicate more concerns. For interventions, current interventions received (e.g. speech therapy, occupational therapy) were reported (0 = no, 1 = yes). The 29-item child health comorbidity survey (0 = never a problem; 1 = problem/concern in past or current; e.g. headaches, dental problems, anxiety, ADHD) was given at ATN Registry baseline. The total number of comorbidities was used. Internal consistency reliability was good in the current study (α = 0.78).

### Statistical Analysis

There were two major study aims: (1) to examine the PAM-DD psychometrics, both as a Guttman scale and as a raw scale; (2) to identify demographic, child, parent, and treatment factors related to activation. Descriptive statistics, frequencies, and percentages were used to describe child and family characteristics.

For aim one, we first verified that the PAM-DD was unidimensional. Item analyses using principal component exploratory factor analysis with varimax rotation were conducted on the 13 raw item scores. Internal consistencies were calculated for the total score and for factors (i.e. subscales) identified by the factor analysis. We also verified the Guttman scaling item weights using item frequencies. Item frequencies should be ordered consistent with the assumed Guttman scaling. Those endorsing items corresponding to the highest levels of activation should endorse items assessing lower levels of activation at even higher frequencies. That is,  endorsement frequencies should be highest for items assessing lower levels of activation and lowest for items assessing higher levels of activation. As described below, our analyses identified four viable activation measures (Guttmann total scale, raw total scale, raw factor 1 subscale and raw factor 2 subscale).

Analyses for aim 2 were conducted in two steps. First Pearson correlations were calculated to identify significant bivariate associations between predictors and the four viable activation measures. Second, multiple regressions were conducted separately with each activation measure as the dependent variable. Variables significantly correlated bivariately with the respective activation measure were included as independent variables.

## Results

### Sample Characteristics

The study sample characteristics, including child and caregiver demographics are shown in Table 1. In the initial enrollment to the RCBA, 784 individuals either declined or could not be reached. A total of 658 families consented and completed the first annual visit for the RCBA. Families who participated in the RCBA study did not differ substantially from families who were recruited from the ATN registry study but declined enrollment.

**Table 1:** Sample characteristics

Parent variables	
Parent relationship to child (%)	
Mother	81.9%
Father	5.7%
Others	1.7%
Did not share	10.7%
Primary caregiver education (%)	
High school or less	13.7%
Some college	26.7%
College and above	48.5%
Missing	11.1%
Income (%)	
$0-$24,999	14.4%
$25,000-$49,999	18.1%
$50,000-$74,999	14.3%
$75,000-$99,999	11.5%
$100,000 +	22.3%
“don’t wish to provide” or “unable to collect”	12.4%
Insurance (%)	
Public Insurance	62.9%
Private Insurance	45.4%
No Insurance	4.1%
Child variables	
Child age, in months, Mean (SD)	72.02 (39.40), range 24–202
Child sex (%)	
Male	80.3%
Female	19.7%
Ethnicity (%)	
Hispanic or Latino Origin	10.2%
Non-Hispanic or non-Latino origin	85.7%
Race (%)	
White	76.7%
Non-white	19.5%
Clinical variables (Visit 1)	Mean (SD)
Child Health and psychological comorbidities (baseline)	4.94 (3.62)
Number of therapies	2.86 (1.77)
Parental concerns	7.05 (3.15)
Sleep problems	44.88 (9.00)
Above sleep problem cutoff (> = 41), %	58.7%
AIM	190.69 (45.74)
Caregiver strain	2.5 (0.77)
PedsQL	63.58 (16.09)
ABC	
Irritability	11.62 (9.41)
Lethargy	8.94 (7.43)
Stereotypy	4.84 (4.63)
Hyperactivity	16.54 (10.96)
Inappropriate speech	3.54 (3.04)
CBCL	
Internalizing behaviors	59.23 (10.27)
Externalizing behaviors	54.64 (11.17)
PAMDD	
Guttman scale	67.36 (10.87)
Raw total	42.15 (5.82)
Vineland	
Adaptive behavior	71.36 (14.69)

The average age of the child participants at the time of ATN enrollment was 72 months (*SD* = 39.40). The majority were male (80%), White (77%), non-Hispanic (86%), and utilized public insurance (63%). Close to half of caregiver participants had college or greater education (49%), and 48% had an annual income of $50,000 or more. Children with ASD in this sample had about five comorbidities (*M* = 4.94; *SD* = 3.62) at ATN enrollment. At the time of the first RCBA visit, children with ASD were receiving about three therapies (*M* = 2.86, *SD* = 1.77), and the total number of parental concerns was about 7 (*M* = 7.05, *SD* = 3.15).

### The Psychometrics, Guttman Scale, and Unidimensionality of the PAM-DD

The PAM-DD mean score was 67.36 (*SD* = 10.87) out of a possible maximum score of 100 when using Guttman scaling, and 42.15 (*SD* = 5.82) out of a possible maximum score of 52 when using raw scoring. Using Guttmann scaling, very few participants (1.2%) were classified at Level 1 (disengaged and overwhelmed) or Level 2 (8.4%; becoming aware but still struggling) activation levels; instead the vast majority were classified at Level 3 (50.8%; ready for action) or Level 4 (25.4%; maintaining behavior and pushing further). The Guttman scaling score was positively and strongly related to the raw PAM-DD total score (*r* = 0.85, *p* < 0.001).

Item frequencies were examined to verify the Guttman item weights. To simplify the results, the 4-point Likert scale was dichotomized (Strongly Agree and Agree = 1; Strongly Disagree and Disagree = 0). Participants reported consistently high activation levels across the items; more than 80% of participants selected Strongly Agree or Agree for all 13 items (see Table 2). In addition, less than 20% of the participants selected Strongly Disagree or Disagree for any items. As shown in Table 2, item frequency rank ordering was inconsistent with the presumed Guttmann scaling. For example, item 9 (“I know what treatments are available for my child’s behavior and development”) had the highest frequency of disagreement (18.7%), followed by items 12 (17.7%; “I am confident I can figure out solutions when new situations arise with my child’s behavior and development), and 11 (17.5%; “I know how to prevent problems with my child’s behavior”).

**Table 2:** PAMDD factor analysis factor loading, item frequency, and actual statement ranking based on item frequency

Item		Factor 1	Factor 2	Item Frequency on Strongly Agree (%)	Actual Ranking
1	I believe that I am that person who is responsible for taking care of my child’s behavior and development	.115	**.542**	98	**2**
2	Taking an active role in my child’s behavioral and developmental needs is the most important thing that affect his/her developmental outcomes,	.190	**.563**	97.7	**3**
3	I am confident I can prevent or reduce problems associated with my child’s behavior and development	.**561**	.320	83.6	10
4	I know what each of my child’s medications are for	.206	**.536**	98.6	**1**
5	I am confident that I can tell when I need to get services for my child and when I can handle my child’s behavior and development	.**496**	.304	90.2	7
6	I am confident I can tell my services provider concerns I have about my child, even when he or she does not ask	.336	**.454**	96.4	**4**
7	I am confident that I can follow through on behavioral and developmental treatments I need to do for my child at home	**.579**	.368	93.9	6
8	I understand the nature and possible causes of my child’s behavior, developmental, or academic concerns	**.570**	.325	86.2	9
9	I know what treatments and strategies are available for my child’s behavior and development	**.545**	.294	81.3	13
10	I have been able to implement recommendations to help my child maintain behavioral and development needs	**.589**	.283	94.8	5
11	I know how to prevent problems with my child’s behavior	**.684**	.193	82.5	11
12	I am confident I can figure out solutions when new situations arise with my child’s behavior and development	**.789**	.141	82.3	12
13	I am confident I can help my child maintain changes (progress), even during times of stress	**.758**	.154	86.4	8

As a further verification check that the actual vs. presumed item rank ordering was consistent with Guttman scaling, each item was assigned an actual rank based on the sample percent that agreed with the statement and a presumed rank based on the item number (e.g. items 1, 2 and 3 were assigned presumed ranks of 1, 2, and 3, and actual ranks of 2, 3 and 10). The correlation between presumed and actual ranks should be 1.0 if the items followed a true Guttman scaling. However, for our data, the correlation between actual and presumed ranks was only 0.63, indicating a substantial departure from cumulative scaling.

In addition to examining the Guttman weights, we performed an EFA using the 13 raw scored items to confirm scale unidimensionality. The factorability of the items was confirmed using multiple criteria. All tests indicated that the scale items met criteria for factorability (Williams et al., 2010). The Kaiser–Meyer–Olkin measure of sample adequacy was 0.90, and Barlett’s test of sphericity was significant (*χ*^*2*^ (78) = 1964.23, *p* < 0.05). In addition, the communalities were all above 0.30. A principal components factor analysis with Varimax (orthogonal) rotation was performed. Two factors emerged and were retained as indicated by eigenvalues greater than 1 and the asymptotic breakpoint for the scree plot (see Table 2). The final 2-factor solution explained 51% of the total variance. The first factor (Eigenvalue = 5.40) accounted for 35% of the variance and included items related to the motivation and ability to actively intervene such as ability to reduce problems, handle services, implement home treatments, understand behavior causes, use available treatments, implement recommendations, prevent problems, figure out solutions, and maintain changes. The second factor (Eigenvalue = 1.28) explained 17% of the variance and contained items related to parental knowledge, cooperation and agreement with treatment, such as responsibility for child’s behavior, participation with treatment role, knowledge about reasons for medication use, and ability to communicate concerns. The internal consistency for the subscales was adequate to good; 0.88 for Factor 1 and 0.66 for Factor 2. As noted earlier, composite scores were created for each factor, with higher scores indicating greater activation.

Correlations between the four methods of assessment activation were examined. Guttman scaled PAM-DD was strongly related to both raw PAM-DD total score (*r* = 0.85, *p* < 0.001) and Factor 1 PAM-DD subscale score (*r* = 0.90, *p* < 0.001), and was modestly related to Factor 2 PAM-DD subscale score (*r* = 0.37, *p* < 0.001). Raw PAM-DD total score was very strongly related to Factor 1 PAM-DD subscale score (*r* = 0.93, *p* < 0.001) and moderately strongly related to Factor 2 PAM-DD subscale score (*r* = 0.68, *p* < 0.001). Finally, the Factor 1 and Factor 2 subscales were modestly related to each other (*r* = 0.36, *p* < 0.001).

### Factors Related to Parent Activation

#### Cross-Sectional Correlational Analyses: Bivariate

Table 3 displays cross-sectional correlations at baseline between the demographic, child, parent and treatment variables and the Parent Activation measures. For most variables, the data were collected during the RCBA visit 1, however, a few variables not expected to change over time were extracted from data collected at the initial enrollment into the ATN registry (e.g. race, gender, ethnicity).

**Table 3:** Correlations Between Demographic, Child, Parent and Treatment Variables and Four Measures of Parent Activation at RCBA Visit 1

	PAM-DD Guttman	Raw PAM-DD	Factor1	Factor2
Caregiver gender	0.05	0.06	0.06	0.02
Caregiver education	− 0.04	0.00	− 0.01	0.01
Income	0.01	0.02	− 0.02	0.08
Private insurance	− 0.01	0.01	− 0.02	0.06
Public insurance	0.07	0.04	0.05	0.00
No insurance	− 0.03	0.01	0.03	− 0.04
Age at registry	− 0.03	0.03	− 0.01	.08*
Participant gender	− 0.01	0.03	0.01	0.04
Race	− 0.06	− 0.02	− 0.03	0.02
Ethnicity	0.03	0.00	− 0.01	0.02
Total comorbidities	0.004	.09*	0.04	.14**
Sleep problems	− .11**	− 0.03	− .09*	.11**
Peds quality of life	.15**	.09*	.15**	− 0.07
ABC irritability	− .12**	− .08*	− .14**	0.07
ABC lethargy	− .08*	− 0.02	− 0.07	.10*
ABC stereotypy	− 0.05	0.00	− 0.04	.09*
ABC hyperactivity	− 0.06	− 0.02	− 0.07	.09*
ABC inappropriate speech	0.02	0.07	0.05	0.08
Internalizing behaviors	− .16**	− .09*	− .15**	0.07
Externalizing behaviors	− .21**	− .14**	− .21**	0.06
Parental concerns	− .12**	− 0.04	− .11**	.12**
Therapy received	− 0.07	0.02	− 0.04	.11**
Adaptive behavior	.11*	.10*	.17**	− .087*
AIM	− 0.01	0.04	− 0.01	.11*
Caregiver strain	− .19**	− .15**	− .23**	0.07

^**^Correlation is significant at the 0.01 level (2-tailed)

^*^Correlation is significant at the 0.05 level (2-tailed)

Except for Factor 2, the results were mostly similar across the activation measures. For example, six of the child and caregiver predictors showed similar patterns of correlations with three Parent Activation measures (Guttmann scaling activation, raw total score activation and Factor 1 activation). Specifically, for all three measures, activation was consistently higher when child irritability (*r*’s = − 0.12, − 0.08, − 0.14), internalizing problems (*r*’s = − 0.16, − 0.09, − 0.15), externalizing problems (*r*’s = − 0.21, − 0.14, − 0.21), and caregiver strain (*r*’s = − 0.19, − 0.15, − 0.23) were lower and when pediatric quality of life (*r*’s = 0.15, 0.09, 0.15) and adaptive behavior were higher (*r*’s = 0.11, 0.10, 0.17), *p* < 0.05.

Similarly, sleep problems and number of parental concerns showed identical patterns of correlations across the four activation measures. Both were negatively related to Guttmann scaling (*r*’s = − 0.11, − 0.12, *p* < 0.01) and Factor 1 activation (*r*’s = − 0.09, − 0.11, *p* < 0.05), but positively related to Factor 2 activation (*r*’s = 0.11, 0.12, *p* < 0.01), and non-significantly related to raw total activation (*p* > 0.05).

In contrast, as noted above, Factor 2 activation was uniquely correlated to several variables either in terms of the direction, e.g. Factor 2 was negatively related to adaptive behavior (*r* = − 0.09, *p* = 0.038) and positively related to sleep problems (*r* = 0.11, *p* = 0.009), or significance of the association. That is, Factor 2 was the only activation measure significantly positively related to older child age at registry (*r* = 0.08, *p* = 0.048), increased levels of child stereotypy (*r* = 0.09, *p* = 0.033), hyperactivity (*r* = 0.09, *p* = 0.024), and autism treatment impact (*r* = 0.11, *p* = 0.013) and increased reception of child therapies (*r* = 0.11, *p* = 0.008).

#### Cross-Sectional Regression Analyses: Multivariate

Four separate multiple regressions were performed using each activation measure as a dependent variable. Predictors entered into the regression were variables significantly correlated to the specific activation measure bivariately. With the exception of Factor 2, there were similarities in the regression results across the different activation scoring methods. Specifically, lower levels of externalizing problems (*ß* = − 0.16, *p* = 0.001; *ß* = − 0.10, *p* = 0.034; *ß* = − 0.12, *p* = 0.009) and decreased caregiver strain (*ß* = − 0.12, *p* = 0.009; *ß* = − 0.14, *p* = 0.004; *ß* = − 0.16, *p* = 0.001) were consistent multivariate predictors of Guttmann scaling activation, raw total activation, and Factor 1 activation. Higher levels of comorbidities (*ß* = 0.12, *p* = 0.005; *ß* = 0.13, *p* = 0.002) were predictors of raw total activation and Factor 2 activation. Factor 1 activation was uniquely related to higher levels of adaptive behaviors (*ß* = 0.11, *p* = 0.011). Other factors were not significant predictors. See Table 4.

**Table 4:** Regression of Demographic, Child, Parent and Treatment Variables and Four Measures of Parent Activation at RCBA Visit 1

	*Beta*	*p*
Predictors PAMDD Guttman Scoring		
V1 externalizing behaviors	− 0.16	0.001
V1 caregiver strain	− 0.12	0.009
*F* (2, 508)		
Predictors of PAMDD Raw Total Scoring		
V1 caregiver strain	− 0.14	0.004
V1 total comorbidities	0.12	0.005
V1 externalizing behaviors	− 0.10	0.034
*R*^*2*^ = .05, *F* (3, 526) = 8.26, p = .000*F* (3, 526)*F* (3, 526)*F* (3, 526)		
Predictors of Factor 1 PAM-DD subscale scoring		
V1 externalizing behaviors	− 0.12	0.009
V1 adaptive behaviors	0.11	0.011
V1 caregiver strain	− 0.16	0.001
*R*^*2*^ = .08, *F* (3, 519) = 15.25, p = .000*F* (3, 519)*F* (3, 519)*F* (3, 519)		
Predictors of Factor 2 PAM-DD subscale scoring		
V1 total comorbidities	0.13	0.002
*R*^*2*^ = .03, *F* (2, 520) = 8.11, p = .000*F* (2, 520)*F* (2, 520)*F* (2, 520)		

## Discussion

This is the first study to characterize parent activation in ASD in a large nationwide sample cross sectionally. Because so little is known about parent activation of children with ASD, our questions concerned not only psychometric validation of different approaches for scoring the PAM-DD but also demographic, child, parent, and treatment correlates of activation. The PAM is based on Guttman scaling or cumulative scaling, and is intended for unidimensional measurement. However, few studies have examined its psychometric properties, factor structure, and presumed Guttman scaling. This study explored the assumptions of the Guttman scaling. Results revealed both consistencies and discrepancies across the four activation scores (i.e. Guttman, raw total, Factor 1, and Factor 2 scores). Several general findings emerged.

The first general finding is that the PAM-DD exhibited several potential problems from a psychometric perspective. Of these, probably the most important was that the PAM-DD does not appear to meet the criteria of a Guttman scale. Analysis of the assumptions of the Guttman scaling revealed contradictory results both in terms of the presumed hierarchical item ordering and presumed unidimensionality. Specifically, the actual ordering of the scale items was inconsistent with the assumptions of a Guttman scale. For example, Item 4, not Item 1 (“I know what each of my child’s medications are for”) recorded the highest frequency of agreement (98.6%), and item 9, not item 13, (“I know what treatments and strategies are available for my child’s behavior and development”) recorded the lowest level of agreement (81.3%). Moreover, the correlation between actual and presumptive item ordering was relatively low for a Guttman scale (*r* = 0.64), indicating that 60% of the variance in item order did not correspond to the assumptions of cumulative scoring, suggesting that the Guttman approach is not being fully realized in this sample.

A related finding is that the PAM-DD failed to clearly meet the assumption of unidimensionality. Factor analysis revealed two reliable factors, which supports the existence of not one, but two dimensions or components. The first factor, which accounted for about a third of the variance, reflected items such as ability to reduce problems, handle services, implement home treatments, understand behavior causes, use available treatments, implement recommendations, prevent problems, figure out solutions, and maintain changes, and mostly concerned taking an active role. The second factor, which explained about 17% of the variance, represented items related to parental knowledge, cooperation and agreement with treatment, such as responsibility for child’s behavior, participation with treatment role, knowledge about reasons for medication use, and ability to communicate concerns, and mostly concerned positive attitudes, cooperation and knowledge.

Finally, the PAM-DD, when scored as a Guttman scale or as a raw scale appeared to be insensitive to measuring the full continuum of activation. Specifically, the vast majority of the sample were classified at the highest, active levels. Fewer than 10% of participants were classified at the two lowest levels, Levels 1 and 2, about 50% were classified at Level 3, and a quarter at Level 4. When individual items were examined, more than 80% of participants endorsed agree or strongly agree for all 13 PAM-DD items. These findings suggest two potential problems when using the scale: (1) possible ceiling effects, i.e. higher levels of activation are being missed, and (2) insensitivity to detecting change, e.g. there is no possibility of detecting positive change for the 25% of the sample categorized at Level 4. Another possibility is that the sample was unrepresentative and highly biased toward high levels of activation.

These findings concerning the problematic psychometrics of the Guttman scored PAM-DD confirmed our decision to further explore the dimensionality of the PAM-DD using exploratory factor analysis. The factor analysis produced two interpretable, unique but correlated factors that helped to extend our understanding of activation. In particular the factors produced quite different results in terms of correlates of activation. The key to understanding these results is that the answers varied depending on which of the two components of activation were examined. There were two general findings.

The first general finding was related to activation as measured by scales highly correlated to Factor 1. Specifically, correlations were similar across the Guttman, raw total and Factor 1 activation scales both in terms of direction of the association and in terms of significant predictors, and for these activation measures (Guttman, raw total, Factor 1), the less challenging the child’s behaviors, the higher the parent activation. The sole contradiction to this general pattern was that total raw score activation was related to increased number of comorbidities. The interpretation of this general result is complicated, however. It could indicate that (a) caregivers of children with fewer challenging behaviors leads to increased feelings of ability and parental activation, (b) that higher activation leads to more help-seeking behaviors that in turn leads to fewer challenging behaviors or (c) that both occur together in some bidirectional, reciprocal fashion. These findings are consistent with Ruble et al. (2019) who found that parents with higher activation also reported increased ability to self-manage their child and decreased parent stress. Future studies are needed to examine the potential directionality using a longitudinal approach. In addition, the duration between receiving the ASD diagnosis and assessing parent activation could help understand some of the findings; however, due to unavailability of this information within the dataset, we were not able to examine this potential explanation.

The second general finding is that Factor 2 tended to be unique in terms of how it was related to the other activation measures and to the predictors. That is, Factor 2 is measuring something slightly different about activation that adds to our understanding of what activation is as a construct. For example, Factor 2 either was uncorrelated to predictors that were significantly correlated to the other activation measures, correlated with predictor variables that were unrelated to the other activation measures or correlated to predictors in the opposite direction. For example, with respect to direction, Factor 2 activation was lower with higher levels of child adaptive behavior and higher with more child sleep problems. With respect to uniquely significant findings, Factor 2 activation was higher when the child was older at initial ATN registry, when the child was rated as displaying more stereotypy, hyperactivity, and higher impact of autism treatment, and when the child received more therapies. Unlike the prior three activation measures, then, the general pattern for this factor is that the more challenging the child’s symptoms, the greater the Factor 2 activation. The multiple regression results confirmed the correlational results. That is, Guttman, raw total, and Factor 1 activation produced similar results, and Factor 2 results were contradictory.

As with most studies, this one suffered from several limitations. First, as noted above, one explanation for some of the contradictory findings is that families receiving services at an ATN site are not necessarily representative of the general autism population because they may be closer to an ATN site geographically. In addition, those families participating in the RCBA study likely represent parents that are more activated than non-participants. That is, the sample was skewed toward highly activated parents, possibly due to the persistent efforts required to become enrolled and obtain services in the ATN, which potentially limits the generalizability of the study. Second, the sample was highly educated (nearly 50% had college degrees). Paradoxically, however, the great majority reported being on public insurance (63%). Another limitation, common to most studies in ASD, was the very high percentage of parental respondents who were mothers (82%). As a result, we cannot be certain that our findings with respect to parent activation levels and predictors will extend to other caregivers (e.g. fathers). A further caveat is that for many of the correlational results, the effect sizes were relatively small. Lastly, experiment-wide Type I error may be increased due to the multiple tests.

In sum, our findings are consistent with DeCamp et al., (2016, 2019) and Liberman et al. (2018) that suggest caution with use of the PAM. The results indicate that the PAM-DD is not unidimensional and consists of two factors in this sample. As already noted, Factor 1 activation was similar to Guttman and raw total activation, whereas Factor 2 activation displayed differences with respect to the other activation measures in the cross-sectional analyses, often with contradictory results. Factor 1 activation, along with Guttman and raw total activation, was higher with less challenging behaviors; the opposite was true for Factor 2 activation. This suggests, at a minimum, that treating the PAM-DD as unidimensional is inadvisable, and that doing so may obscure our ability to uncover more nuanced findings. Factor 2 activation seems most consistent with parental knowledge, cooperativeness and agreement with treatment, and with more passive, but supportive, involvement, whereas Factor 1 activation implied taking a more active role in treatment. However, further research will be needed to fully understand and untangle these somewhat contradictory results.
